# Transcranial Doppler and Magnetic Resonance in Tanzanian Children With Sickle Cell Disease

**DOI:** 10.1161/STROKEAHA.118.018920

**Published:** 2019-06-14

**Authors:** Edward N. Kija, Dawn E. Saunders, Emmanuel Munubhi, Angela Darekar, Simon Barker, Timothy C.S. Cox, Mechris Mango, Deogratias Soka, Joyce Komba, Deogratias A. Nkya, Sharon E. Cox, Fenella J. Kirkham, Charles R.J.C. Newton

**Affiliations:** 1 From the Muhimbili Wellcome Programme, Dar es Salaam, Tanzania (E.N.K., E.M., D.S., J.K., D.A.N., S.E.C., C.R.J.C.N.); 2 Muhimbili University of Health and Allied Sciences, Dar es Salaam, Tanzania (E.N.K., M.M., D.S., C.R.J.C.N.); 3 Developmental Neurosciences and Biomedical Research Unit, UCL Great Ormond Street Institute of Child Health, United Kingdom (D.E.S., T.C.S.C., F.J.K.); 4 Clinical and Experimental Sciences, University of Southampton, United Kingdom (F.J.K.); 5 University Hospital Southampton, United Kingdom (A.D., S.B., F.J.K.); 6 London School of Hygiene and Tropical Medicine, United Kingdom (S.E.C.); 7 Nagasaki University School of Tropical Medicine and Global Health, Japan (S.E.C.); 8 Department of Psychiatry, University of Oxford, United Kingdom (C.R.J.C.N.).

**Keywords:** brain, hemoglobin, infarction, magnetic resonance angiography, magnetic resonance imaging

## Abstract

**Background and Purpose—:**

We determined prevalences of neurological complications, vascular abnormality, and infarction in Tanzanian children with sickle cell disease.

**Methods—:**

Children with sickle cell disease were consecutively enrolled for transcranial Doppler; those with slightly elevated (>150 cm/s), low (<50 cm/s) or absent cerebral blood flow velocity (CBFv) were invited for brain magnetic resonance imaging and magnetic resonance angiography.

**Results—:**

Of 200 children (median age 9; range 6–13 years; 105 [2.5%] boys), 21 (11%) and 15 (8%) had previous seizures and unilateral weakness, respectively. Twenty-eight (14%) had elevated and 39 (20%) had low/absent CBFv, all associated with lower hemoglobin level, but not higher indirect bilirubin level. On multivariable analysis, CBFv>150 cm/s was associated with frequent painful crises and low hemoglobin level. Absent/low CBFv was associated with low hemoglobin level and history of unilateral weakness. In 49 out of 67 children with low/absent/elevated transcranial Doppler undergoing magnetic resonance imaging, 43% had infarction, whereas 24 out of 48 (50%) magnetic resonance angiographies were abnormal. One had hemorrhagic infarction; none had microbleeds. Posterior circulation infarcts occurred in 14%. Of 11 children with previous seizure undergoing magnetic resonance imaging, 10 (91%) had infarction (5 silent) compared with 11 out of 38 (29%) of the remainder (*P*=0.003). Of 7 children with clinical stroke, 2 had recurrent stroke and 3 died; 4 out of 5 had absent CBFv. Of 193 without stroke, 1 died and 1 had a stroke; both had absent CBFv.

**Conclusions—:**

In one-third of Tanzanian children with sickle cell disease, CBFv is outside the normal range, associated with frequent painful crises and low hemoglobin level, but not hemolysis. Half have abnormal magnetic resonance angiography. African children with sickle cell disease should be evaluated with transcranial Doppler; those with low/absent/elevated CBFv should undergo magnetic resonance imaging/magnetic resonance angiography.

## Abstract 2

Supplemental Digital Content is available in the text.

Although there are few data available from sub-Saharan Africa, stroke and silent cerebral infarcts (SCI) detected by magnetic resonance imaging (MRI) are common in untreated sickle cell disease (SCD) elsewhere.^1,2^ Arterial ischemic stroke in the territories of the middle and anterior cerebral arteries and their border zones is typical.^3,4^ Posterior reversible encephalopathy syndrome has been reported^5^ and is not always reversible although residual occipital or cerebellar infarction is rarely reported in cross-sectional studies. Hemorrhagic stroke occurs in the context of hypertension^6^ and aneurysm formation,^7^ but the prevalence of microbleeds remains uncertain.

Magnetic resonance angiography (MRA) detects intracranial arteriopathy.^8^ Stenosis or occlusion of the intracranial arteries may be detected with transcranial Doppler (TCD), measuring cerebral blood flow velocity (CBFv); abnormal internal carotid artery (ICA)/middle cerebral artery (MCA) CBFv (>200 cm/s) occurs in 2.8% to 10% of children^9,10^ and predicts clinical stroke.^11^ Low anterior cerebral artery and MCA CBFv may also predict stroke and SCI,^12^ but there is an overlap with those with inadequate TCD;^10^ there are few data comparing TCD with MRA in this group.

Data from Africa on the prevalence in SCD of TCD outside the normal range (50–149 cm/s)^13^ are scarce, although conditional (>170 and ≤200 cm/s) and abnormal CBFv (>200 cm/s) have been reported in 4% and 7%, respectively^14^; there seems to be considerable variability. Of 105 Kenyan children, only 3% had conditional, and none had abnormal CBFv.^15^ A Nigerian study of 145 children found that 5% had CBFv >200 cm/s and 20% had CBFv >150 cm/s,^16^ whereas in Cameroon, 22% had abnormal MCA CBFv.^17^ MRI was not performed in any of these studies.

We conducted a cross-sectional study to determine the prevalence of elevated/absent/low CBFv in Tanzanian children with SCD and imaged those with abnormal TCD to determine whether there was an association between elevated or low CBFv and silent infarction or microbleeds in the anterior or posterior circulation on MRI or cerebrovascular disease on MRA.

## Methods

Ethical clearance and permission to conduct this study were obtained from the Senate Research and Publications Committee of Muhimbili University of Health and Allied Sciences and Muhimbili National Hospital. All parents/guardians of the children gave written consent. The data that support the findings of this study are available from the corresponding author on reasonable request.

The inclusion criteria for the study were children with SCD (confirmed with hemoglobin electrophoresis; no hemoglobin SC disease in this cohort) aged between 6 and 13 years enrolled in the Muhimbili Sickle Cohort^18^ who consecutively attended the SCD outpatient clinic of the Muhimbili National Hospital in Dar es Salaam, Tanzania from June 2010 to March 2011. Penicillin was prescribed, and use of insecticide-treated nets was emphasized to prevent malaria infections, but chloroquine and hydroxyurea were not prescribed, and no child was on a chronic blood transfusion regime. A structured questionnaire was used to determine demographic characteristics, and clinical events and all children had a complete general and neurological examination. We used a recall period of 2 years for admissions and blood transfusions and 1 year for painful crises to minimize bias. Blood pressure, oxygen saturation, and hemoglobin were measured on the day of the TCD examination when the child was well.

The basal cranial arteries (MCA, anterior cerebral artery, ICA) were insonated with a 2 MHz probe and nonimaging TCD (Viasys Health Care). All examinations were performed by 1 author (E.N. Kija), with Dr Newton checking the recordings outside the normal range. The CBFv in the MCA/ICA was classified as follows low (CBFv <50 cm/s), normal (50–149 cm/s),^13^ slightly elevated (150–169 cm/s), conditional (170–199 cm/s), and abnormal (CBFv≥200 cm/s).^11^ Children who had a TCD exam outside the normal range (no signal, CBFv <50 cm/s, or CBFv >150 cm/s) were scheduled to have brain MRI/MRA brain (Figure I in the online-only Data Supplement).

MRI scanning was performed on a Phillips Achieva 1.5 Tesla MRI scanner, using an 8-channel head coil, at a median duration of 5 months (range 3–7 months) after the TCD examination. The scanning protocol included T1-weighted spin echo (repetition time [TR]/echo time [TE]/flip angle=596 ms/15 ms/69°), T2-weighted fast spin echo (TR/TE/flip angle=4424 ms/100 ms/90°), T2* weighted gradient echo (T2*gradient echo; TR/TE/flip angle=700 ms/23 ms/18°), fluid-attenuated inversion recovery (TR/TE/TI/flip angle=11 000 ms/140 ms/2800 ms/90°), diffusion-weighted imaging (*b*=0, 500, 1000 s/mm^2^) and susceptibility weighted imaging (TR/TE/flip angle=35 ms/50 ms/15°), all acquired in the axial plane, and 3D time-of-flight MRA (TR/TE/flip angle=35 ms/5 ms/20° and 0.7 mm slice thickness).

T2-weighted, fluid-attenuated inversion recovery and diffusion-weighted imaging images were used to determine the presence of infarcts and atrophy. Images were reviewed independently by 3 neuroradiologists (D.E. Saunders, S. Barker, T.C.S. Cox); where there was disagreement, the scans were reviewed by Dr Saunders, and consensus was achieved when 2 neuroradiologists agreed.

SCI was defined as ≥1 focal MRI signal abnormalities of at least 3 mm in 1 dimension visible in 2 views on fluid-attenuated inversion recovery/T2-weighted images in the absence of a focal neurological deficit.^2^ T2*gradient echo and susceptibility weighted imaging were inspected for hemorrhage. MRA maximum intensity projections were used to determine the presence of intracranial artery abnormality using a previously reported grading system.^18^

Children were followed until censoring at stroke or death or March 31, 2014.

The data were analyzed in SPSS v22. Skewed data were summarized with median and interquartile range. Association between variables was determined using χ^2^ test and Fisher exact tests. Univariable and multivariable logistic regression were used to determine clinical predictors of elevated/absent/low CBFv. Mann-Whitney and Kruskal-Wallis tests were used to compare the distribution of variables between 2 and >2 variables, respectively. Interobserver variability between the neuroradiologists was analyzed using the method of Fleiss with the web-based programme of Geertzen (https://mlnl.net/jg/software/ira/). A *P*<0.05 was considered as statistically significant.

## Results

From June 2010 to March 2011, 200 consecutive children (median age, 9; range, 6–13 years; 105 (52.5%) boys) meeting the inclusion criteria were enrolled. Fifteen (8%) had a history of unilateral weakness, and 21 (11%) had a history of seizures (Table 1). TCD examination was outside the normal range in 67 (34%) children, of whom 28 (14%) had elevated CBFv >150 cm/s and 39 (20%) had CBFv<50 cm/s (n=10) or absent signal (n=29; Table 1). Sixteen had slightly elevated CBFV (150–169 cm/s), 11 (5.5%) had conditional CBFv (170–199 cm/s), but only one, who had already had a clinical stroke, had an abnormal high CBFv (>200 cm/s). Elevated CBFv was more common in children aged <12 years old. Hemoglobin was lower in those with low/absent and elevated CBFv, but there was no association with indirect bilirubin as a marker of hemolysis (Table 1).

**Table 1.**
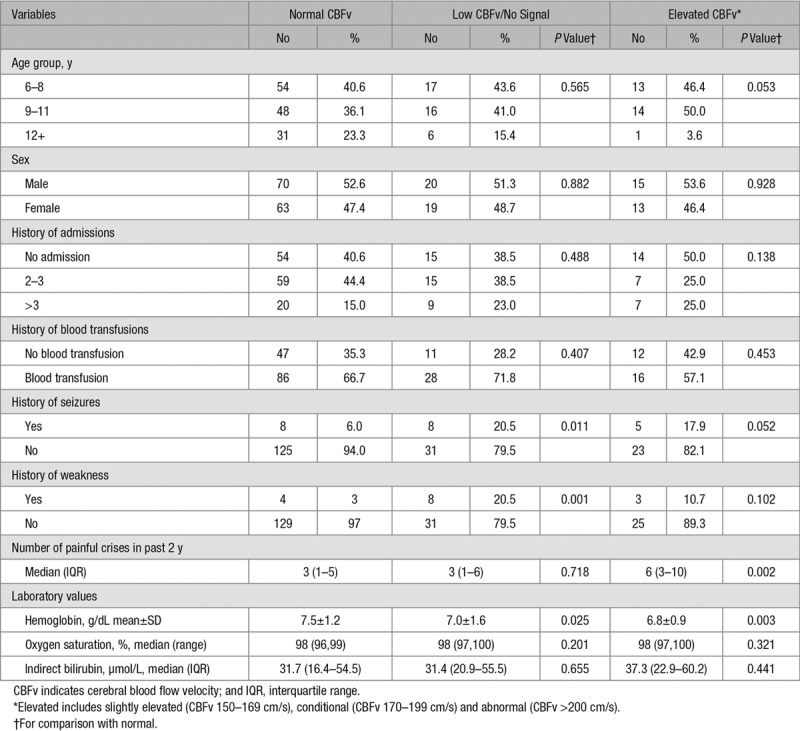
Patient Characteristics by CBFv Category

### Risk Factors for Low CBFv or Absent MCA Signal

Compared with those with normal velocities, children with absent/low CBFv or MCA signal were more likely to have a history of focal weakness and seizures (Table 2). In multivariable analysis, low hemoglobin and history of focal weakness were independent predictors of absent/low CBFv (Table 2).

**Table 2.**
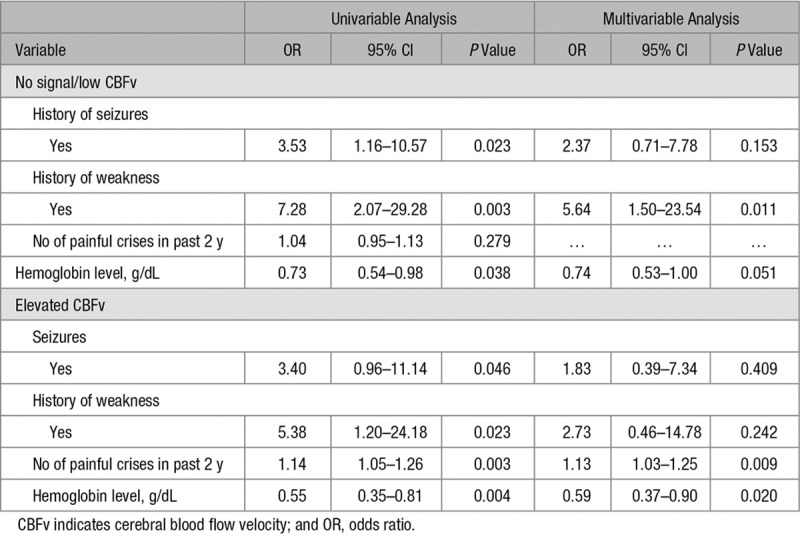
Risk factors for No Signal/Low CBFv and Elevated CBFv

### Risk Factors for Elevated CBFv

Compared with children with normal CBFv, those with elevated CBFv >150 cm/s in the MCA were more likely to have a history of seizures, but not focal weakness. They also had more frequent painful crises in the previous 2 years compared with those with normal CBFv (Table 1). In multivariable analysis, low hemoglobin and frequent painful crises were independent predictors of elevated CBFv (Table 2).

### Patients Who Had MRI Scans

Of the 67 children with TCD examinations outside the normal range, 18 (27%) did not return for MRI (Figure I in the online-only Data Supplement). There was no difference in the demographic or clinical features of those children who did not return for the scans compared with those who did (Table I in the online-only Data Supplement). For documentation of infarction among the 3 observers, κ was 0.629 (good agreement); whereas for determination of normal MRA and definite MRA abnormality, κ was 0.849 (excellent agreement).

**Figure 1.**
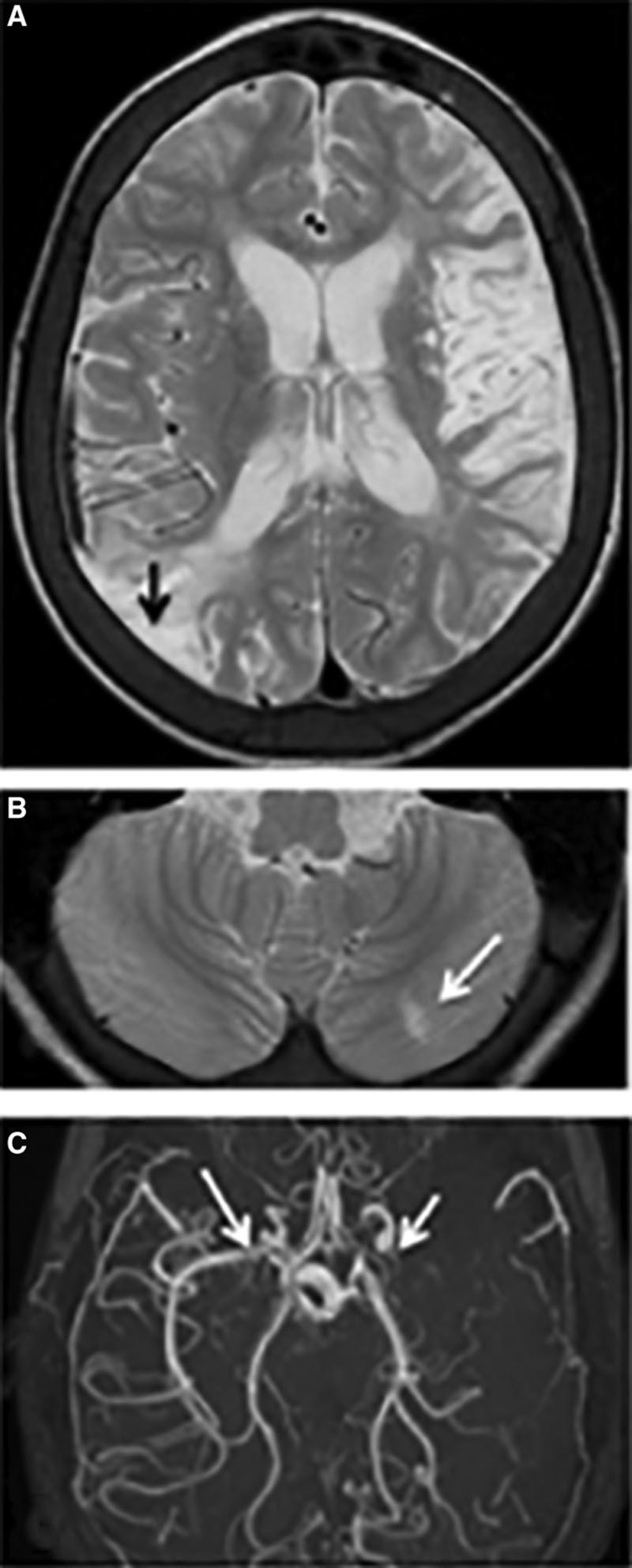
Ten-y-old Tanzanian girl with seizures and focal weakness with absent transcranial Doppler velocities. **A**, Axial T2-weighted magnetic resonance image showing mature right MCA (middle cerebral artery)/PCA (posterior cerebral artery) watershed territory (black arrow) and left MCA territory infarcts. Bilateral deep gray and deep white matter watershed lesions are seen bilaterally. **B**, A small left cerebellar infarct (white arrow) is seen in the same patient. **C**, The magnetic resonance angiogram reveals an occluded left MCA (short arrow) and a narrow right MCA (long arrow). The PCAs appear normal. Marrow expansion of the skull vault is noted. The patient subsequently died.

**Figure 2.**
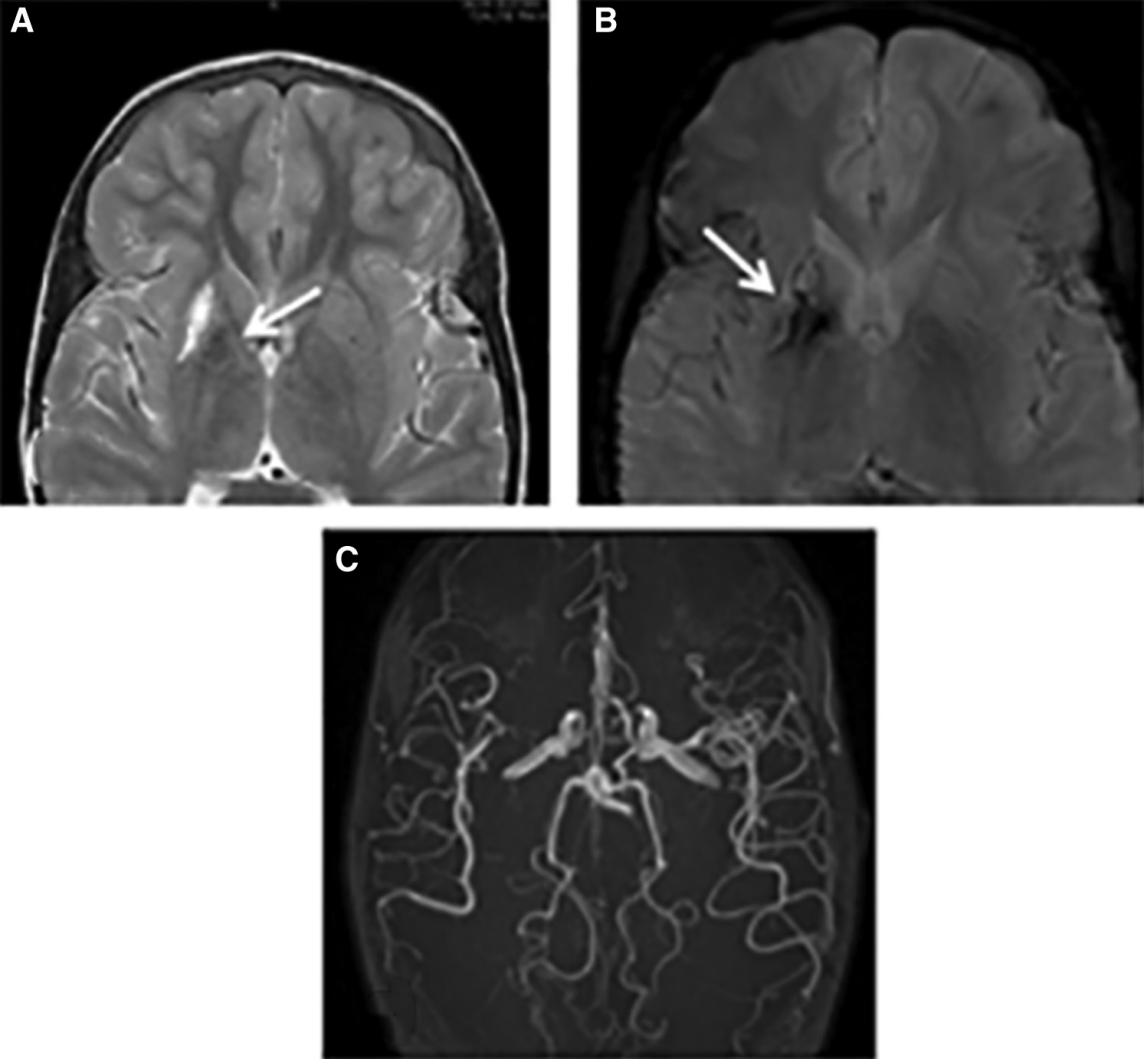
**A**, Hemorrhagic basal ganglia infarct in a 16-y-old girl not reporting symptoms but with asymmetric transcranial Doppler velocities (right time averaged mean of the maximum 39, left 164 cm/sec). The subtle hemorrhagic change is seen as a dark blush on the T2-weighted sequence but is well seen on the (**B**) T2* sequence (white arrows). **C**, A magnetic resonance angiogram revealed severe stenosis of the right middle cerebral artery with reduced filling of the distal vessels. She was followed for 3 y and did not develop neurological symptoms.

Of the 49 children who had MRI, 21 (43%) had abnormal scans (Figures 1 and 2; Figures II and III in the online-only Data Supplement), that is, at least 21 out of 200 (11%) of the overall sample. Most infarcts occurred in the anterior circulation, in deep white matter and basal ganglia (Table 3), but 7 (14%) had occipital or cerebellar infarcts or atrophy (Table 3, Figure 1). All but one had large vessel disease, mostly involving the terminal cavernous and carotid arteries; in 2, the posterior circulation infarction was associated with generalised disease. Seven patients had had clinical stroke (14% of those undergoing MRI, 4% of the total and 70% of those with a history of focal weakness). SCI occurred in 14 (29% of those undergoing MRI or at least 7% of the overall sample). SCI was present in 8 out of 21 (38%) of the children who had CBFv >150 cm/s, and in 6 out of 24 (25%) of the children with absent signal, but there were no SCI in the 4 with low CBFv. One patient with slightly elevated CBFv had hemorrhage associated with basal ganglia infarction (Figure 2), but no microbleeds were detected on T2*gradient echo or susceptibility weighted imaging.

**Table 3.**
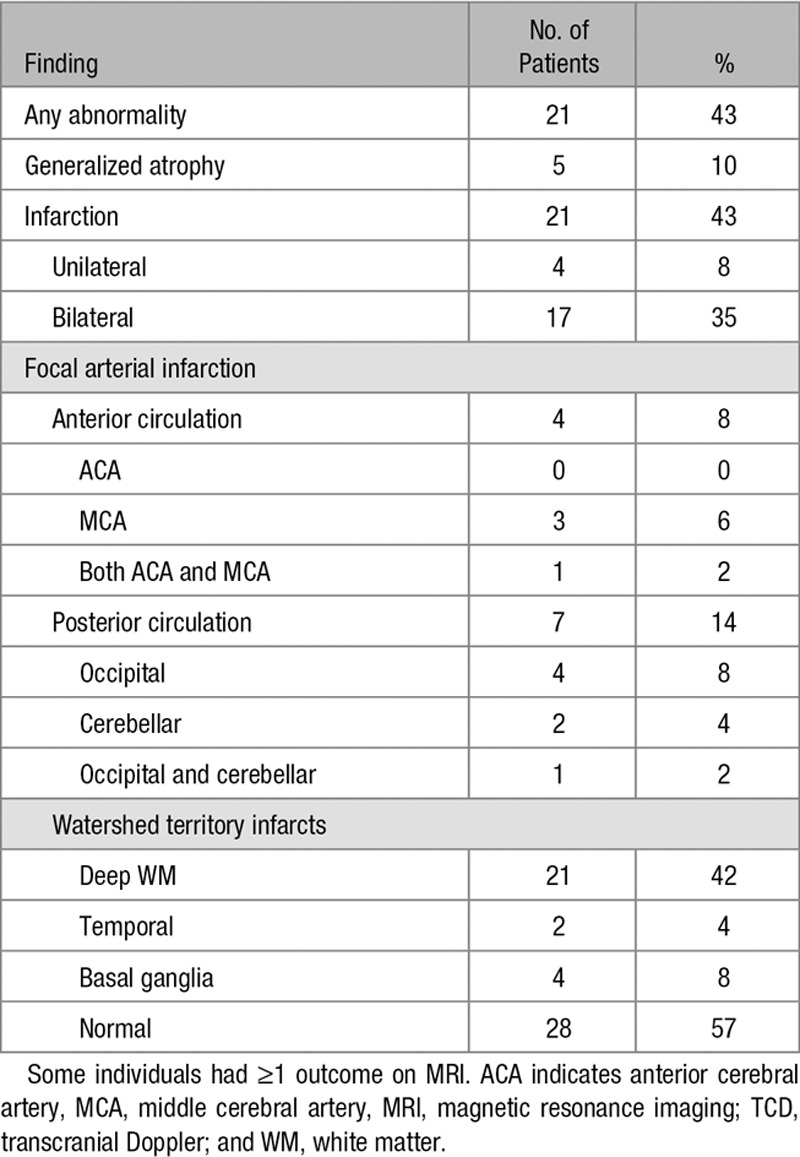
MRI Findings in 49 Children With Abnormal TCD

Twenty-four patients with TCD outside the normal range had abnormal MRA (Figures 1 and 2; Figures II and III in the online-only Data Supplement; Table 4), 50% of the 48 scanned (1/49 with clinical stroke and abnormal CBFv>200 cm/s failed MRA). This represents at least 36% of the 67 with elevated/low CBFv, and the majority had stenosis/occlusion (grades 2/3).^18^ One patient had moyamoya (grade 4;^18^ Figure II in the online-only Data Supplement). Turbulence without definite narrowing or occlusion (grade 1;^18^ Figure III in the online-only Data Supplement) was not counted as abnormal. MRA was more likely to be abnormal in those with infarction on MRI (χ^2^=12.34; *P*=0.00044). Of the 6 out of 7 children with clinical stroke who had MRA, all were abnormal, whereas of the 14 children with SCI who also had MRA, 10 (71%) were abnormal. Abnormal MRA was documented in 8 out of 24 with absent TCD signal (33%), 1 out of 4 with CBFV<50 cm/s (25%) and 9 out of 20 (45%) with CBFV>150 cm/s.

**Table 4.**
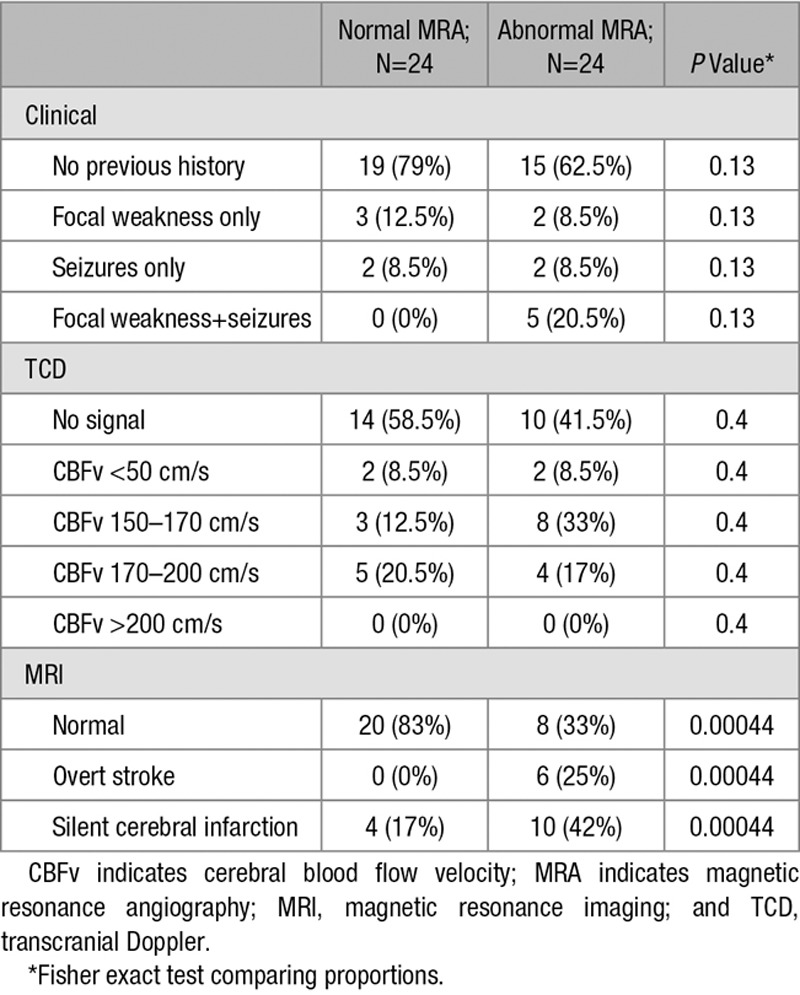
Relationship Between Clinical, TCD, MRI, and MRA Findings

Although abnormal ICA/MCA velocity (>200 cm/s) was rare in this cohort, absent or low velocities were common in those with previous clinical presentations and those with abnormal MRA (Table II in the online-only Data Supplement). Of the 7 with clinical stroke, 2 had elevated and 5 had absent CBFv. Of the 14 with SCI, 8 had elevated CBFv and 6 had absent CBFv. Three patients died, 2 of whom had prior strokes with absent CBFv and 1 with absent CBFv and normal MRI who died during an episode of severe anemia. Four children had a subsequent stroke, 1 with absent CBFv and SCI and 3 with previous stroke (2 with elevated CBFv, 1 with absent CBFv). The remaining 13 children with SCI did not experience subsequent strokes and, although 2 of the 11 with conditional TCD and the 1 child with abnormal TCD had had previous neurological symptoms, these 10 children did not experience stroke during follow-up.

Of 11 children with a history of seizures undergoing MRI, 10 (91%) had infarction (5 silent) compared with 11 out of 38 (29%; 10 silent) of those with no history of seizures (Fisher exact test *P*=0.0003); 7 out of 10 also had MRA abnormality compared with 17 out of 38 of those without seizures (Fisher exact test *P*=0.14). Seven of 9 children (78%) with a history of contralateral weakness had stenosis or occlusion of the basal cranial arteries on MRA compared with 17 out of 39 (44%) of those without weakness (*P*=0.137).

## Discussion

More than a third of Tanzanian children with SCD had TCD recordings outside the normal range (<50 or >150 cm/s), consistent with cerebrovascular disease or, for those with elevated CBFv, cerebral hyperemia. Although 12 (6%) had CBFv>170 cm/s, only one patient (0.5%) had abnormally high CBFv>200 cm/s. Frequent painful crises in the previous year and lower hemoglobin was associated with a CBFv>150 cm/s. Low hemoglobin and focal neurological deficits were associated with low/absent velocities. The proportion of children with elevated, low, or absent CBFv who had infarction on MRI was 43%, with SCI comprising 31% of this selected group.

The prevalence of TCD outside the normal range was similar to most of the previous studies in Africa.^14–17^ The lower proportion of abnormal high CBFv (>200 cm/s) may be because of rapid progression of the disease in Africa, with progressive stenosis of internal carotid and MCAs leading to occlusion with lower CBFv distally, consistent with our finding of a high prevalence of absent/CBFv, particularly in those with clinical presentations. The high mortality for the children with clinical stroke undergoing MRI (2/7) is consistent with the possibility that those with progressive cerebrovascular disease died at a younger age; African survival cohorts may, therefore, seem to have less severe CVD. We documented stenosis and occlusion on MRA, although only one child had moyamoya collaterals. MRA of the neck might reveal additional stenosis or occlusion.^19^

The prevalence of focal weakness was similar to studies of stroke from the pre-TCD screening era in North America,^1^ and the prevalence of seizures is similar to data from Nigeria,^20^ although a little higher than in the Jamaican cohort.^21^ As the patients with elevated/low/absent CBFv were selected for MRI, the minimum prevalence of SCI of 7% is likely to be an underestimate. Although there is a previously documented association between abnormal high CBFv >200 cm/s and SCI, most with SCI have normal TCD.^22^ Our observed SCI prevalence in midchildhood (at least 7% of the total population and 29% of those scanned for elevated/low/absent CBFv), seems lower than that in unselected populations elsewhere.^2^ A larger study of unselected patients will be required to determine whether this holds for those with CBFv within the normal range, as well as those with elevated or low CBFv. This is important as 1 study found that children with normal TCD velocities and no SCI had a lower rate of neurological events compared with those with SCI and a normal TCD.^23^ This is a survival cohort with high prevalence of alpha thalassemia^14^ and Central African Republic haplotypes, which are associated with reduced risk of stroke, so it is biologically plausible that SCI is less common.

The distribution of infarction in the basal ganglia and deep white matter is similar to that reported in other studies,^24^ but we also report occipital and cerebellar infarction typical of a posterior circulation distribution; the anterior cerebrovascular disease associated may reflect the severity of disease in untreated children with SCD. The absence of corresponding posterior large vessel disease in children with posterior circulation infarcts may reflect the lack of imaging of the vertebrobasilar system in this cohort but also raises the possibility of an alternative cause, such as poor oxygen delivery or cardiac embolic source.

Interestingly, abnormal MRA was documented in more than half of the children with CBFv>150 cm/s, including 8 out of 11 with CBFv 150–169 cm/s as well as 4 of 9 with conditional TCD (the child with abnormal TCD failed MRA). Absent/low CBFv in the ICA/MCA may be secondary to extracranial stenosis or occlusion with low distal flow (not excluded as neck vessel imaging was not performed), as well as intracranial disease, demonstrated on MRA in 50% of low and 42% of absent TCD in this study, or to difficulties in obtaining signal through a thick skull.^10^

In our study, all of those with overt stroke, and nearly 3 quarters of those with SCI, had abnormal MRA, apparently higher than in previous US studies. MRA abnormality was reported in 58% of children with clinical stroke in the SWiTCH study (Stroke With Transfusions Changing to Hydroxyurea),^8^ in 16% of those with SCI in the SIT trial (Silent Infarct Transfusion),^22^ and in 25% of children with SCD in the STOP study (Stroke Prevention Trial in Sickle Cell Anemia) of children with abnormal CBFV>200 cm/s.^25^ In the Tanzanian children, the most common MRA abnormality found in children with elevated/low/absent CBFv was stenosis, similar to the studies in the American children.^25^ In contrast to children with stroke, in whom MRA abnormality was only seen in those with absent/low CBFv,^8^ we also documented abnormal MRA in children with CBFv>150 cm/s.

Seizures were more common in children with elevated/absent/low CBFv, consistent with previous UK data.^26^ In our study, children with elevated/low/absent CBFv and a history of seizure had a higher proportion of infarcts than children who did not have a history of seizure. The CSSCD study (Cooperative Study of Sickle Cell Disease) of American children also found that seizures were associated with stroke and SCI,^27^ but did not report TCD or MRA. Prengler et al^26^ found no association between abnormal MRA findings and history of seizure, although CBFv was higher in those with seizures. Cerebrovascular disease should be excluded in children with SCD and seizures, although seizures may occur early in an active pathophysiological process when CBFv is abnormal, but MRA is not.

### Study Limitations

Some of the clinical parameters are prone to recall bias despite the relatively short period. Given the high mortality of children with SCD in Tanzania within the first 5 years of life,^28^ the children with the most severe disease may have died and the data may not be generalizable. MRI and MRA were obtained at a mean of 5 months after the TCD, during which time CBFv may have changed. Children were recruited from outpatient clinics when clinically well, leading to a potential for bias in excluding sicker children which may account for the lack of children with abnormal TCD, although routine outpatients is also typically the setting for TCD screening in the North. As a result of their relative rarity in this cohort, it was neither possible to explore any differences in effect of conditional or abnormal CBFv compared with slightly elevated CBFv nor to determine whether elevated TCD was associated with abnormal MRA. A quarter of children with CBFv outside the normal range did not return for MRI, which may have introduced selection bias, although there was no evidence for selection of children by age or with a history of neurological problems. Children with SCD and normal TCD examinations did not undergo MRI, whereas MRA of the neck was not included, so the proportions of SCI and MRA abnormality are minimum estimates. The prevalence of abnormalities may be different, but our interobserver variability for MRI reporting was good, similar to that reported from the SIT trial^29^ with the same definition^2,30^ and that for the MRA abnormalities was even better.

### Conclusions

This study shows that elevated/absent/low CBFv and MRI abnormalities are common in children with SCD in Africa and that frequent painful crises in the past year and low hemoglobin are independent risk factors for these abnormalities in addition to seizures and clinical stroke. TCD may be useful in centers in Africa that do not have access to MRI, provided that evidence-based treatment for Conditional or Abnormal CBFv is available; there is now some evidence that hydroxyurea, which is relatively low cost, reduces CBFv. Longitudinal studies are required to determine the outcome and neurocognitive functioning of children with TCD outside the normal range and abnormal MRI/MRA.

## Sources of Funding

The Wellcome Trust, UK (Project grant 080025; Strategic award 084538).

## Disclosures

None.

## Supplementary Material
