# Supplementary material for: Transcranial Doppler and Magnetic Resonance in Tanzanian Children With Sickle Cell Disease
Source: Stroke. 2019 Jun 14;50(7):1719–26. doi: 10.1161/STROKEAHA.118.018920 (PMC6594727; doi:10.1161/STROKEAHA.118.018920)
Supplement: Supplementary file 1 [file str-50-1719-s001.pdf]

## SUPPLEMENTAL MATERIAL

### Supplementary figure I: Study Flow diagram

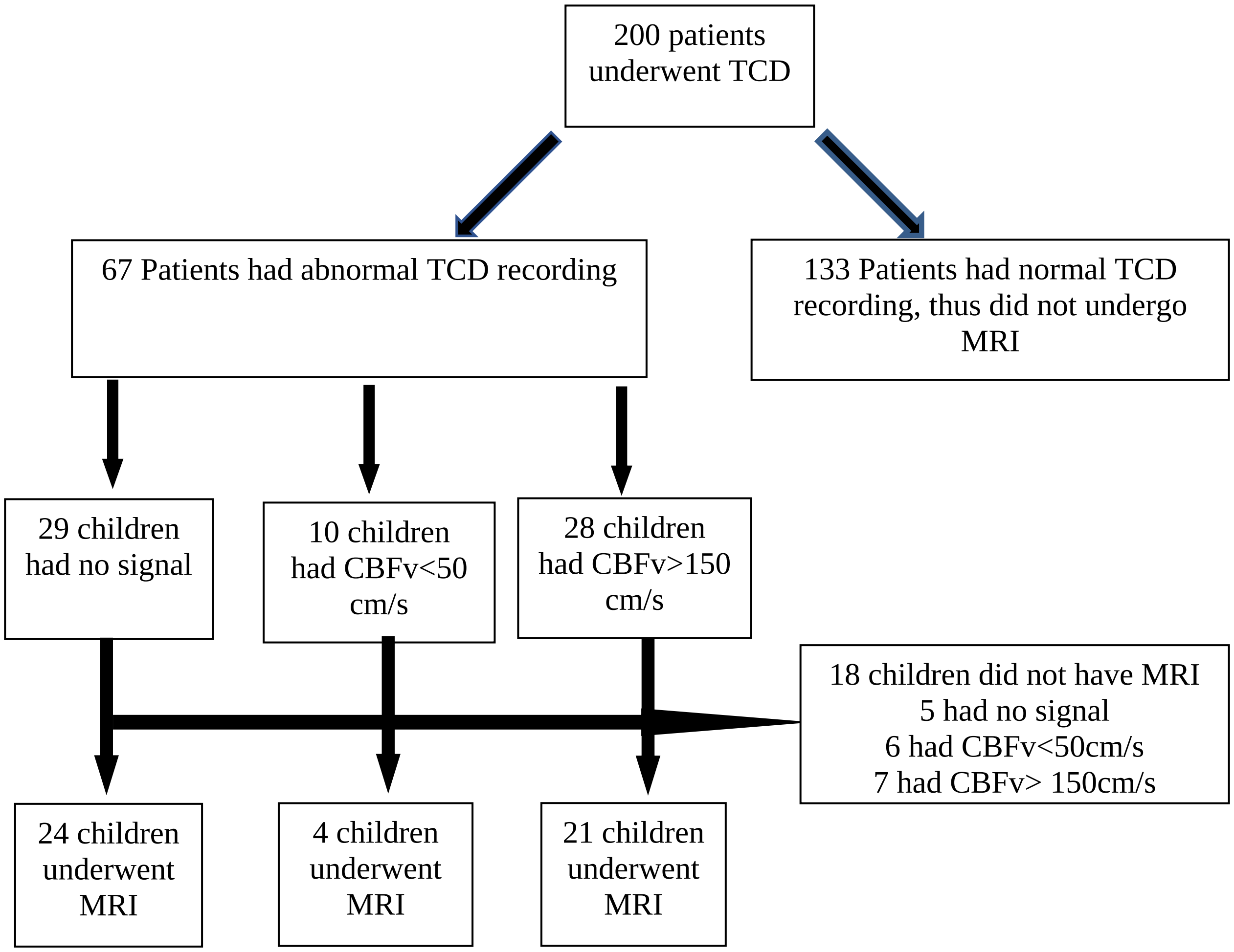

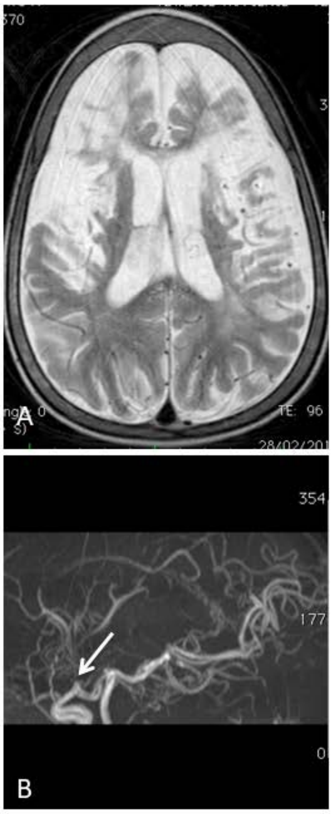


Supplementary Figure II A. Axial T2-weighted image through the basal ganglia reveals mature bilateral MCA infarcts involving the caudate nuclei heads on a background of cerebral atrophy in a 12 year old boy. B. The lateral view of the MRA shows that the terminal ICAs are narrowed to the Pcom arteries where they are occluded (white arrow). The MCA and ACAs fill via moyamoya collaterals and the PCA and its branches supply prominent pial collaterals (*).

AP = anteroposterior, ACA = anterior cerebral artery, MCA = middle cerebral artery, PCA =posterior cerebral artery, Pcom = posterior communicating artery.

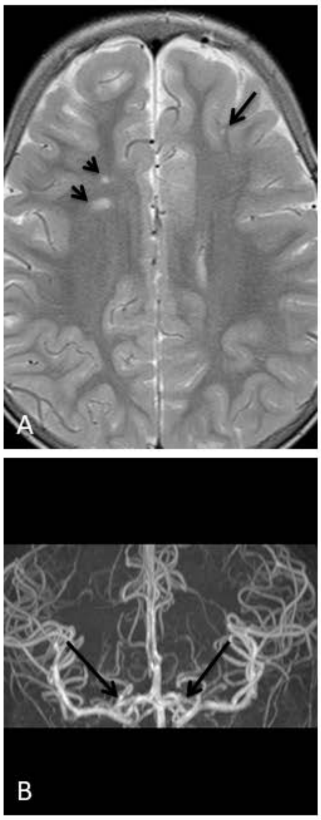


Supplementary Figure III A. Axial T2-weighted image through the centrum semiovale of a 10 year old girl. Deep white matter infarcts (arrowheads) are seen within the right frontal white matter. A small subcortical lesion (arrow) is seen on the left. B. Turbulence is seen in the proximal ACAs of the MRA on a rotated AP view. The remainder of the vessels and branches are well visualised with normal appearing calibre and flow.

AP = anteroposterior, ACA = anterior cerebral artery, MCA = middle cerebral artery, PCA =posterior cerebral artery, Pcom = posterior communicating artery.

**Supplementary Table I: Demographics and clinical features of children with cerebral blood flow velocity (CBFv) outside the normal range who did and did not undergo magnetic resonance imaging (MRI)**

| | | MRI (n=49) | No MRI (n=18) | p |
|---|---|---|---|---|
| CBFv | Elevated<br>Reduced | 21<br>28 | 7<br>11 | 1.0 |
| Age group | <8 years<br>9-11 years<br>>12 years | 22<br>20<br>7 | 8<br>10<br>0 | 0.2 |
| Sex | Female<br>Male | 26<br>23 | 6<br>12 | 0.2 |
| History of focal weakness | Yes<br>No | 10<br>39 | 2<br>16 | 0.5 |
| History of seizures | Yes<br>No | 11<br>39 | 2<br>16 | 0.5 |

**Supplementary Table II: Relationship between Clinical history, MRI and MRA, and Middle Cerebral Artery Velocity**

| | Right MCA CBFv | | | | | | Left MCA CBFv | | | | | |
|---|---|---|---|---|---|---|---|---|---|---|---|---|
| | No signal N=19 | Low (<50) N=3 | Normal (50-149) N=17 | High (150-170) N=4 | Conditional (>170<200) N=6 | Abnormal (>200) N=0 | No signal N=24 | Low (<50) N=2 | Normal (50-149) N=9 | High (150-170) N=9 | Conditional (>170<200) N=4 | Abnormal (>200) N=1 |
| **Clinical history** | | | | | | | | | | | | |
| None N=34 | 10 | 3 | 14 | 4 | 3 | 0 | 14 | 2 | 7 | 8 | 3 | 0 |
| Seizures N=5 | 2 | 0 | 1 | 0 | 2 | 0 | 3 | 0 | 2 | 0 | 0 | 0 |
| Focal weakness (stroke/TIA) N=4 | 2 | 2 | 0 | 0 | 0 | 0 | 3 | 0 | 0 | 0 | 1 | 0 |
| Stroke/TIA + seizures N=6 | 5 | 0 | 0 | 0 | 0 | 1 | 4 | 0 | 0 | 1 | 0 | 1 |
| **Infarction on MRI (N=49)** | | | | | | | | | | | | |
| Normal N=28 | 10 | 2 | 10 | 2 | 4 | 0 | 14 | 2 | 7 | 3 | 2 | 0 |
| Silent (covert) infarction N=14 | 4 | 0 | 7 | 2 | 1 | 0 | 6 | 0 | 2 | 5 | 1 | 0 |
| Overt stroke N=7 | 5 | 1 | 0 | 0 | 1 | 0 | 4 | 0 | 0 | 1 | 1 | 1 |
| **MRA (N=48)** | | | | | | | | | | | | |
| Normal N=24 | 9 | 1 | 9 | 2 | 3 | 0 | 14 | 1 | 4 | 3 | 2 | 0 |
| Abnormal N=24 | 10 | 2 | 8 | 2 | 2 | 0 | 10 | 1 | 5 | 6 | 2 | 0 |
